# Glycyrrhizin could reduce ocular hypertension induced by triamcinolone acetonide in rabbits

**Published:** 2011-08-04

**Authors:** Zhengyu Song, Yuanyuan Gong, Haiyun Liu, Qiushi Ren, Xiaodong Sun

**Affiliations:** 1 Shanghai First People’s Hospital, School of Medicine, Shanghai JiaoTong University, Shanghai, P.R. China; 2 Beijing Universitiy, Beijing, P.R. China

## Abstract

**Purpose:**

To evaluate the hypotensive effects of glycyrrhizin (GL) on a rabbit model of ocular hypertension (OH) induced by triamcinolone acetonide (TA).

**Methods:**

Forty New Zealand White Rabbits were divided as follows: control (intravitreal injection of sterile saline solution); GL (intravitreal injection of sterile saline solution, then fed with 25mg GL/day); TA (intravitreal TA injection); TA+GL (intravitreal TA injection, then fed with GL) and GL+TA (pre-treated with GL for 3 days, then got TA injection and the following GL treatment). Intraocular pressure (IOP), flash electroretinogram (flash ERG) and flash visual evoked potential (flash VEP) were measured during the follow-up (28 days). The aqueous humor was analyzed, using ^1^H-nuclear magnetic resonance spectroscopy and principal components analysis (PCA).

**Results:**

IOP elevation was observed in the TA group during the follow-up, compared to the controls (p<0.01). The IOP was decreased in the TA+GL group and the GL+TA group, compared to the TA group (p<0.05). Both in flash ERG and VEP, the amplitudes were decreased, and the implicit time was prolonged in the TA group, compared to the controls (p<0.05); and the parameters were improved after intervention of GL, compared to the TA group (p<0.05). PCA results indicated that TA could affect ocular metabolism (especially the sugar metabolism), and GL could inhibit it.

**Conclusions:**

The administration of GL could suppress OH induced by TA in rabbits, and improve their electrophysiological parameters. Metabolomics is a useful tool in ophthalmology research. Our results indicate that TA-induced ocular metabolism changes could be compensated by GL.

## Introduction

Corticosteroid induced glaucoma (CIG) is a kind of secondary open angle glaucoma occurred in susceptible person after general or topical administration of glucocorticoid (GC) [1]. Ticho et al. [2] found that dexamethasone could lead to abnormal accumulation of acid mucopolysaccharide in the chamber angle. Some researchers [3-5] reported that 3 alpha, 5 beta-tetrahydrocortisol (steroid antagonist) could lower the intraocular pressure (IOP) in steroid induced ocular hypertension (OH) cases. But the pathogenesis of CIG/OH still remains unclear, and the drug therapy has limited effects. The incidence of CIG/OH has increased gradually by widely using of triamcinolone acetonide (TA) and GC-containing eye preparations in recent years. It is reported that 30%–62.3% of patients have experienced CIG/OH up to 24 months after intravitreal injection of TA [6-8]. About 0.3%–3.3% of patients had to perform anti-glaucomatous surgery or laser therapy (selective laser trabeculoplasty, etc.) because of uncontrolled OH [7,8]. Even after the surgery, some patients still had permanent loss of visual acuity and impairment of the visual field [9]. So the targeted therapy is urgently needed. New methods, including anecortave and gene therapy virus (GC-inducible MMP1), are reported to be effective in animal models [10-12]. However, those methods are invasive, and may have severe side effects (endophthalmitis, hemorrhage, etc.).

In vivo, there exists a GC balance, which includes cortisone (no biologic activity)/ cortisol (biologic activity) [13]. As a low affinity NADP (H)-dependant enzyme with bi-direction (11-oxo-reductase and dehydrogenase), 11β-hydroxysteroid dehydrogenase type 1 (11β-HSD1) is a tissue-specific regulator of GCs [13-15]. Mainly acting as a reductase in ocular tissue, 11β-HSD1 can transform cortisone into cortisol, and then cortisol can increase the resistance of aqueous humor outflow to raise IOP [13,16,17]. So 11β-HSD1 is regarded as a potent target to regulate GC activity. A significant reduction (10%–20%) of IOP after the systemic administration of carbenoxolone (CBX), a non-selective inhibitor of 11β-HSD1 and used to treat digestive ulcer, could be found in normal volunteers (especially from day 3 to day 7) [16,17]. And Rauz et al. [18] reported those who had a fall in IOP also demonstrated a change in steroid metabolites consistent with 11β-HSD1 inhibition. But CBX is out of use because of severe complications (hypertension, electrolyte disturbance, etc.). Glycyrrhizin (GL), oral administration to treat liver diseases (liver cirrhosis and so on), can be transformed into glycyrrhetinic acid (GA) in vivo [19]. GA can inhibit 11β-HSD1 in liver and kidney, with little mild complications [20,21]. It was reported that 5β-dihydro-cortisol could enhance the function of cortisol in eye [22], both GL and CBX can potently inhibit 5β-reductase. But there have been no studies associated with GL in CIG/OH yet, as far as we know.

The pathogenesis of CIG/OH still remains unclear and it may involve many cross-linking cytokines, signaling pathway and biochemical changes, which are all closely related to the GC metabolism. Metabolomics (or metabonomics) is a quantitative measurement of 'dynamic multi-parametric metabolic responses to pathophysiological stimuli or genetic modification in living systems' [23,24]. It has been widely used in the evaluation of drugs (toxicity, effect, mechanism, and indication). Its major methods include nuclear magnetic resonance spectroscopy (NMR) and mass spectrometry. NMR data, combined with multivariate statistical analysis (such as Principal components analysis, PCA), is a useful tool to investigate the pathophysiological metabolism [25,26]. However, to the best of our knowledge, there have been no metabolomics researche associated with CIG/OH or GCs as yet.

The purpose of this study was to evaluate the ocular hypotensive effects of GL on a rabbit model of OH induced by TA, and evaluate the metabolic changes of aqueous humor using ^1^H-NMR spectrum coupled with PCA. Flash electroretinogram (flash ERG) and flash visual evoked potential (flash VEP) was also performed to estimate the visual function.

## Methods

### Rabbits

Forty New Zealand White Rabbits (NZWR) of male, weighing 2.0 to 2.2 kg, were treated in accordance with the Association for Research in Vision and Ophthalmology (ARVO) Statement for the Use of Animals in Ophthalmic and Vision Research. Only right eyes were used in this study. To exclude the presence of any pre-existing abnormalities, the eyes were examined by slit-lamp and indirect ophthalmoscope. Blood pressure was recorded every day, and blood biochemistry test was performed pre and at 1, 7, and 28 days post-injection.

The environment for animal raising was set up as follows: light (7AM-7PM)/dark (7PM-7AM) cycle; temperature (22–25 °C); humidity (55%–60%); feeding two times (100 g standard feed for each time) per day (8AM, 6PM); no limits to water and activity.

The rabbits were divided into five groups randomly (8 eyes in each group). In the control group, the eyes were injected with 0.1 ml sterile saline solution (BSS; Alcon China Ophthalmic Product Company Ltd., Beijing, China) intravitreally; in the GL group, the eyes were injected with 0.1 ml BSS intravitreally first, then fed with half Glycyron Tablets (Minophagen Pharmaceutical Co., Ltd., Tokyo, Japan) per feeding in the following 28 days; in the TA group, the eyes were injected with 0.1 ml/4 mg TA (Bristol-Myers China Squibb Company, Shanghai, China) intravitreally; in the TA+GL group, the eyes were injected with 0.1 ml/4 mg TA first, then received the same GL treatment as in the GL group; in the GL+TA group, the rabbits were fed with GL 3 days before TA injection, then got the same treatment as in the TA+GL group.

### Anesthesia

Rabbits were anesthetized via intramuscular injection of a mixture containing pentobarbital (20 mg/kg body wt) and ketamine hydrochloride (20 mg/kg body wt) before the injection, measurement of IOP, testing for flash ERG and flash VEP, or withdraw of aqueous humor. Topical anesthesia (2% lidocaine hydrochloride) was administered to reduce discomfort.

### Injection

Before TA injection, the drug was pretreated according to Bitter et al. [27] to remove preservatives. Intravitreal TA injection was performed 2.0 mm posterior to the limbus in the superotemporal quadrants under visualization, using a 27 G needle. And after the injection, the port was pressed with a cotton swab for 30 s to prevent backflow.

### IOP measurment

IOP measurement was performed pre-injection and at 1, 7, 14, 21, and 28 days post-injection, using a Tono-pen tonometer (Reichert, Inc., Depew, NY). Each eye was measured three times and the mean data were recorded.

### Flash ERG and flash VEP test

All rabbits were examined by RETI port21 (Roland Retiscan, Wiesbaden/Brandenburg, Germany) pre-injection and 7, 14, and 28 days post-injection. Before test, pupils were fully dilated using a mixture of 0.5% tropicamide and 0.5% phenylephrine hydrochloride, then the rabbits were dark-adapted for 30 min before scotopic flash ERG examination, and light-adapted for 2 h before photopic flash ERG test (background light intensity was 33 cd). A unipolar contact lens was placed on the cornea with goniosol (IOLAB Corporation, Claremont, CA). The reference electrode (stainless steel needle) was placed on the subcutaneous space of the forehead, and ground electrode was clipped to the earlobe. Flash ERG signals were amplified (×20,000) and filtered (0.3–300 Hz) by differential amplifiers with a maximum intensity (5.76 cd·s/m^2^). The implicit time (IT) and amplitude of flash ERG signals were automatically measured by a computer program.

Flash VEP signals were recorded using a stainless steel needle, as the active electrode, inserted under the skin above the area of the visual cortex midway between two ears. The reference and ground electrodes were inserted in the ears. The signals were amplified (×500,000) and filtered (1–100 Hz) by differential amplifiers. The IT and amplitude of the second negative peak (N2) were automatically measured by a computer program.

### Aqueous humor acquirement

After penetrating into anterior chamber with a 27 G needle in the temporal limbus, 0.1 ml aqueous humor was acquired pre- and at 1 and 28 days post-injection. The samples were immediately snap-frozen and stored at −30 °C.

### NMR spectroscopy

The samples were centrifuged at 12,000× g for 10 min at 4 °C to isolate the precipitate. Then the supernatants were moved into 5 mm NMR tubes, which contained 100 μl D_2_O for field frequency lock. All NMR spectra were recorded on a Bruker AVANCE III 600 NMR spectrometer (Bruker BioSpin GmbH, Rheinstetten, Germany) operating at 599.69 MHz (^1^H-frequency) at 25 °C. Standard one-dimensional PRESAT spectra were acquired using a single 90° pulse sequence. All spectra were phase- and baseline-corrected with reference to the methyl peak of lactate (CH_3_, 1.33).

### Data reduction and pattern recognition

All NMR spectra were data-reduced to 241 integrated regions of equal width of 0.04 ppm (buckets) corresponding to the region of δ10.0 to 0.4, by VNMR 6.1C software package (Varian Inc., Los Angeles, CA). The remaining spectral segments for each NMR spectrum were normalized to the total sum of the spectral intensity to partially compensate for differences in concentration of the many metabolites in the samples. Subsequently, the normalized integral values were input into SIMCA-P 10.5 software package (Umetrics, Umeå, Sweden) as variables and were mean centered for the analysis of PCA. Data were visualized with the principal component (PC) scores plot of the first two principal components (PC1 and PC2) to provide the most efficient 2D representation of the information contained in the data set, where each point represents an individual spectrum of a sample.

### Statistics

Data were presented as means±SD. The results were analyzed by the ANOVA (ANOVA, SPSS 11.5, SPSS Inc., Chicago, IL). The difference was considered significant when p<0.05.

## Results

### General condition

The eyes were examined by means of the slit lamp and indirect ophthalmoscope during the follow-up. No retinal detachment and endophthalmitis were noted in the experimental eyes. No apparent abnormality was found in blood pressure and blood biochemistry test.

### IOP

There were no significant differences in IOP in any of the groups pre-injection (p>0.05). IOP in the GL group was lower than that in the control group during the follow-up (p<0.05). Significant elevation of IOP was noticed in the TA group at 1, 7, 14, 21, and 28 days post-injection, compared to the controls (p<0.01). IOP in the TA+GL group was lower than that in the TA group during the follow-up (p<0.01), except at 1 day post-injection (p*=*0.602). There were no significant differences in IOP between the TA+GL group and the GL+TA group in the follow-up (p>0.05), except higher IOP was recorded in the TA+GL group at 1 day post-injection (p*=*0.004). The IOP changes in this experiment are shown in Table 1.

**Table 1:** IOP changes in rabbits after administration of GL (mm Hg).

**Group division**	**Pre**	**1d**	**p**	**7d**	**p**	**28d**	**p**
Control	15.5±2.3	16.2±2.4	<0.001	15.8±2.2	<0.001	16.4±2.9	0.035
GL	15.8±3.2	15.4±4.5	<0.001	14.6±3.6	<0.001	15.1±3.1	<0.001
TA	15.7±2.8	37.3±3.7	0.602	39.4±3.5	<0.001	37.5±3.8	<0.001
TA+GL	15.4±2.6	38.5±2.7		28.2±5.6		26.8±6.3	
GL+TA	15.7±3.5	30.1±4.9	0.004	27.3±4.7	0.523	26.1±5.7	0.782

p, the p-value (ANOVA) when the TA+GL group was compared to other groups.

### Flash ERG and flash VEP

No significant differences in IT or amplitudes were found in any of the groups pre-injection (p>0.05). In flash ERG, the amplitudes were decreased, and IT was prolonged in the TA group during the follow-up, compared to the controls (p<0.05). The amplitudes were increased, and IT was decreased in the TA+GL group and the GL+TA group during the follow-up, compared to the TA group (p<0.05). There were no significant differences between the TA+GL group and the GL+TA group in the follow-up (p>0.05). In flash VEP, the amplitudes were decreased, and IT was prolonged in the TA group at 28 days post-injection, compared to the controls (p<0.05). The amplitudes of the TA+GL group and the GL+TA group were higher, and IT was lower than those in the TA group at 28 days post-injection (p<0.05). There were no significant differences between the TA+GL group and the GL+TA group (p>0.05). Flash ERG and flash VEP changes in this experiment are shown in Table 2, Table 3, and Table 4.

**Table 2:** Amplitudes of flash ERG in rabbits after administration of GL (μv).

**Group division**	**Time**	**Rod-R**	**Max-R**		**OPS**	**Cone-R**		**30 Hz-F**
** **		**b wave**	**a wave**	**b wave**	**total**	**a wave**	**b wave**	**Amp**
**Control**								
** **	Pre	182.4±31.3	100.6±23.3	220.6±37.1	156.7±30.3	27.4±4.2	105.6±22.1	31.6±5.4
** **	7d	177.4±29.4	97.3±22.5	215.1±30.6	152.3±27.6	26.5±5.1	100.3±23.1	30.7±6.3
** **	28d	174.6±33.3	95.0±20.8	212.4±43.7	149.4±26.9	24.9±5.6	96.2±25.8	28.8±4.7
**GL**								
** **	Pre	180.3±40.1	102.4±34.2	224.3±32.5	153.2±36.2	26.9±3.7	104.1±24.3	31.4±4.8
** **	7d	174.6±31.5	99.2±23.4	218.1±37.4	152.6±24.8	26.5±4.3	101.2±26.7	30.2±5.9
** **	28d	172.7±34.1	97.4±21.2	213.2±36.8	150.3±28.6	25.3±4.4	98.4±24.5	27.9±3.2
**TA**								
** **	Pre	186.4±34.5	98.1±25.9	226.7±36.3	160.2±28.3	26.4±4.6	102.6±28.7	32.8±5.6
** **	7d	158.4±27.2	82.4±21.7	200.6±32.7	140.9±25.2	21.9±3.6	84.6±20.3	27.7±5.1
** **	28d	152.2±28.3	79.7±19.2	190.4±28.6	133.7±29.3	19.7±3.7	79.2±19.3	24.9±4.3
**TA+GL**								
** **	Pre	189.4±35.2	96.5±24.6	225.6±44.2	162.4±32.5	27.9±4.9	100.4±29.5	31.2±5.3
** **	7d	179.3±22.2**	94.7±25.4**	221.5±38.3**	158.5±19.3**	25.8±3.9**	95.3±24.8**	30.5±6.4**
** **	28d	164.2±19.8*	86.3±18.6*	203.9±37.4*	140.8±24.2*	22.5±4.8*	89.0±26.8*	26.3±3.7*
**GL+TA**								
** **	Pre	186.1±33.2	95.1±34.5	222.7±41.6	163.3±36.9	27.2±4.7	101.5±26.3	30.8±6.4
** **	7d	176.2±31.2**	96.3±22.2**	218.4±35.6**	157.3±21.8**	26.1±3.6**	96.4±22.7**	30.8±5.3**
** **	28d	165.7±23.2*	89.6±20.6*	205.7±35.2*	143.6±25.4*	22.9±3.3*	88.1±24.7*	25.8±3.6*

*, p<0.05 (ANOVA) when the TA group compared to other groups. **, p<0.01 (ANOVA) when the TA group was compared to other groups.

**Table 3:** Implicit time of flash ERG in rabbits after administration of GL (ms).

**ERG parameters**	**Control**			**TA**			**TA+GL**		
** **	**Pre**	**7d**	**28d**	**Pre**	**7d**	**28d**	**Pre**	**7d**	**28d**
Rod-R	46.3±4.8	46.7±4.2	47.2±3.9	46.4±3.3	50.2±4.3	50.7±4.5	45.2±3.7	45.9±4.4**	48.8±3.8*
Max-R	38.1±5.2	38.7±4.9	39.2±4.3	38.5±4.2	42.8±5.3	43.1±2.3	37.8±3.5	38.2±4.1**	40.9±5.7*
Cone-R	27.8±3.7	28.4±4.2	28.6±3.1	27.5±4.7	33.2±4.6	34.0±4.7	27.6±3.6	27.7±4.4**	31.2±4.9*

*, p<0.05 (ANOVA) when the TA+GL group compared to other groups. **, p<0.01 (ANOVA) while the TA+GL group compared to the TA group.

**Table 4:** Amplitudes (baseline=100) and Implicit time (ms) of flash VEP in rabbits after administration of GL.

**VEP parameters**	**Control**			**TA**			**TA+GL**		
** **	**Pre**	**7d**	**28d**	**Pre**	**7d**	**28d**	**Pre**	**7d**	**28d**
IT	24.9±2.6	24.2±3.2	25.5±3.9	25.2±3.6	25.0±3.4	27.6±3.8**	24.6±3.1	24.9±2.9	26.8±2.3*
Amplitude	100	93.2±18.9	104.7±14.3	100	92.7±15.8	77.2±13.4**	100	96.3±14.8	89.7±13.6*

*, p<0.05 (ANOVA) when the TA+GL group compared to other groups. **, p<0.01 (ANOVA) when the TA group compared to the control group.

### NMR spectroscopy

**Figure 1:**
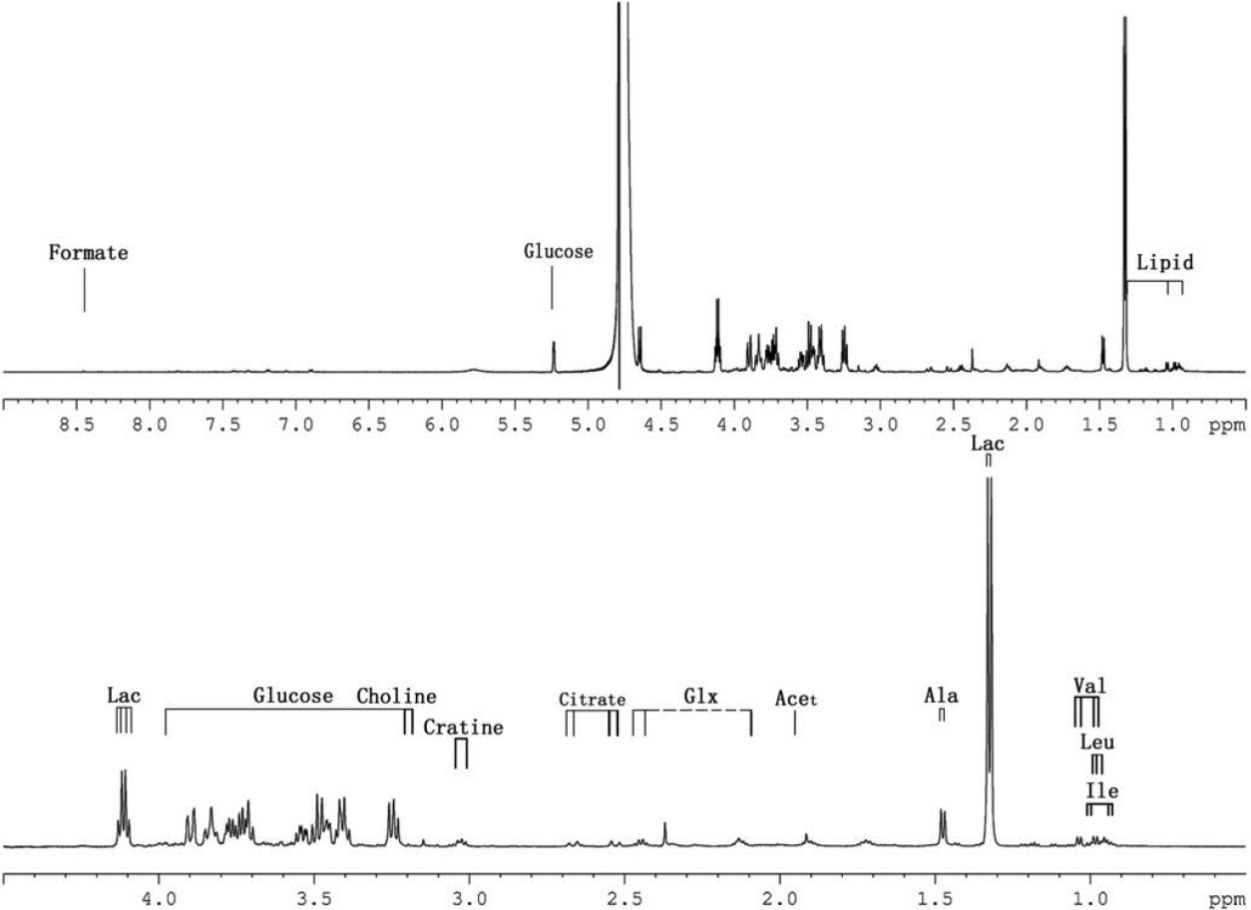
Typical 600 MHz ^1^H-NMR spectrum (0–9 ppm) of rabbit aqueous humor from the TA+GL group (one day post-injection).

A total of 18 metabolites, based on their characteristic chemical shifts and multiplicities, were identified in ^1^H-NMR spectrum within the region from 0 to 9 ppm. Those metabolites included isoleucine, leucine, valine, alanine, lactate, acetate, glutamine and glutamate complex (Glx), citrate, trimethyamine, creatine, N-acetylglycoproteins, choline, taurine, glycerol, glucose, fatty acid (low density lipoprotein, very low density lipoprotein) and formate. Typical ^1^H-NMR spectrum of aqueous humor was shown in Figure 1.

### PCA

No significant differences in metabolites were found in any of the groups pre-injection.

The concentration of 12 metabolites (low density lipoprotein, very low density lipoprotein, isoleucine, leucine, valine and N-acetylglycoproteins increased; alanine, lactate, choline, taurine, glycerol and glucose decreased) were significantly changed in the aqueous humor of NZWR in the TA group at 1 day after intravitreal injection, compared to the controls. Six significantly changed metabolites (alanine, lactate, acetate, taurine, glycerol and glucose) were found in the TA group at 28 days post injection, compared to the controls. Compared the NMR spectrum in the TA group at 1 day with 28 days, seven metabolites (low density lipoprotein and very low density lipoprotein decreased; alanine, lactate, taurine, glycerol and glucose increased) were changed.

There were 7 changed metabolites (lactate, choline, taurine and glucose increased; low density lipoprotein, very low density lipoprotein and N-acetylglycoproteins decreased) in the aqueous humor of NZWR in the TA+GL group and the GL+TA group at 1 day post-injection, compared to the TA group. The same metabolic changes were found in the TA+GL group and the GL+TA group at 28 days post-injection, except N-acetylglycoproteins.

There were no significant metabolites changes between the GL+TA group and the TA+GL group, except higher levels of lactate and glucose in the GL+TA group at 1 day post-injection.

**Figure 2:**
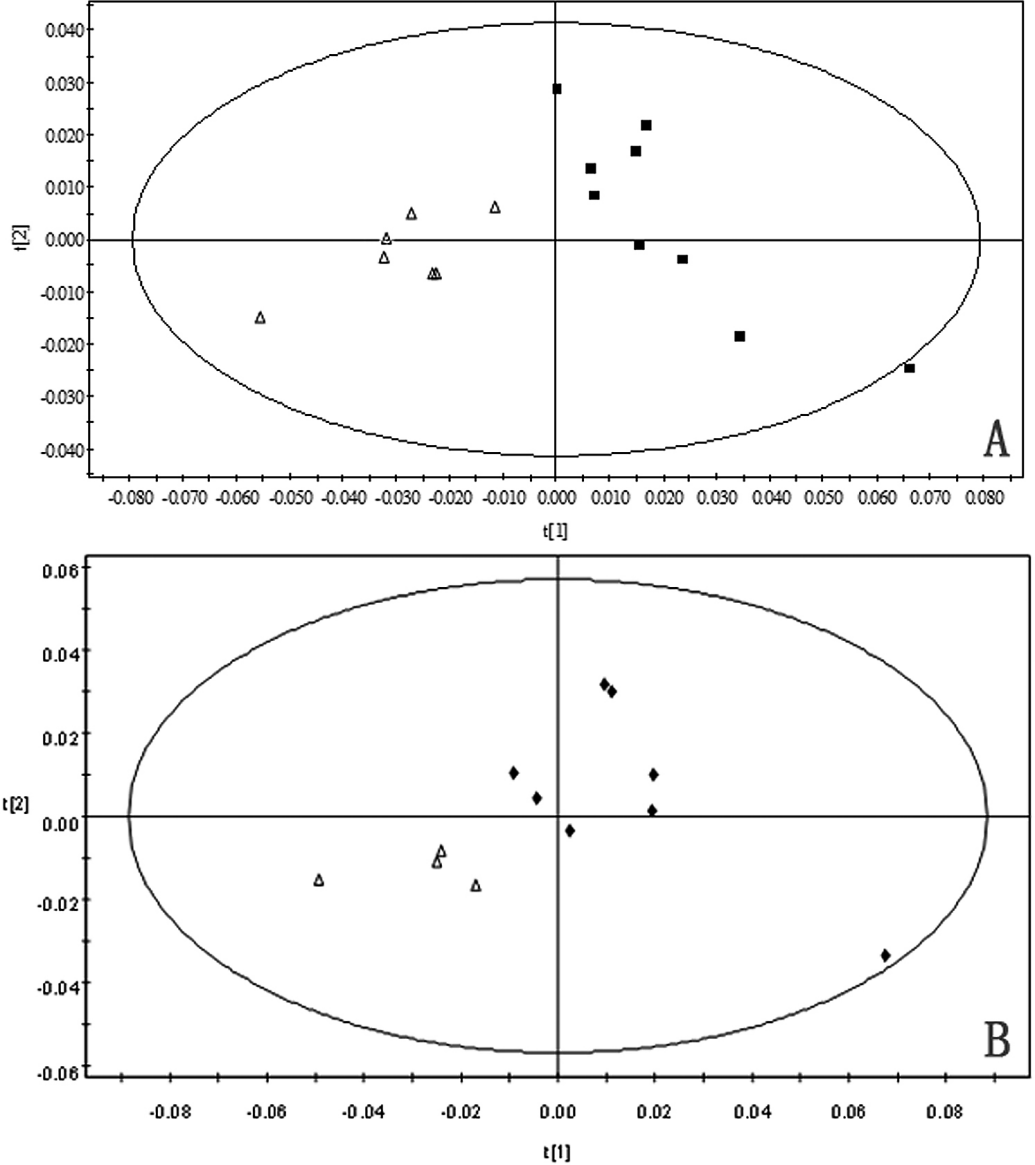
The confidence ellipse of PCA scores plots. **A**: PCA scores plots of 1H-NMR spectrum between the TA group (◇) and the TA+GL group (■), one day post-injection. **B**: PCA scores plots of 1H NMR spectrum between the TA group (◆) and the TA+GL group (△), 28 days post-injection.

The PCA results are shown in Table 5 and Figure 2.

**Table 5:** Comparison of metabolites concentration in rabbits after administration of GL by PCA.

**Group division **	**LDL**	**VLDL**	**Isoleucine**	**Leucine**	**Valine**	**Alanine**	**Lactate**	**Acetate**	**N-AGP**	**Choline**	**Taurine**	**Glycerol**	**Glucose**
TA versus control (d1)	↑	↑	↑	↑	↑	↓	↓		↑	↓	↓	↓	↓
P	0.033	0.032	0.023	0.018	0.024	0.015	0.015		0.000	0.008	0.006	0.034	0.034
TA versus control (d28)						↑	↑	↓			↑	↑	↑
P						0.017	0.014	0.035			0.009	0.032	0.000
TA+GL versus TA (d1)	↓	↓					↑		↓	↑	↑		↑
P	0.028	0.021					0.026		0.002	0.007	0.000		0.013
TA+GL versus TA (d28)	↓	↓					↑			↑	↑		↑
P	0.017	0.013					0.031			0.012	0.000		0.000
TA+GL versus control (d28)	↓	↓				↑	↑	↓					↑
P	0.034	0.027				0.024	0.013	0.021					0.019
GL+TA versus TA+GL (d1)							↑						↑
p							0.015						0.000

LDL, Low-density lipoproteins; VLDL, Very low-density lipoproteins; N-AGP, N-acetylglycoproteins. ↑, Increase; ↓, Decrease; Blank, No change.

## Discussion

Glycyron is a first-line drug for hepatic cirrhosis and other liver diseases, with little side effects. Each tablet contains 25 mg GL and Supplemental Materials (25 mg glycine and 25 mg D-L-methionine). And in this experiment, it showed no side effects in the rabbits by blood pressure and blood biochemistry testing.

The ocular hypotensive effects of GL on a rabbit model with OH were evaluated in this experiment. Our data showed that IOP in the TA+GL group and the GL+TA group was significantly lower than that in the TA group. Both GL pretreatment and post-treatment had the same hypotensive effects, except that GL pretreatment had better IOP control at 1 day after intravitreal TA. Those results indicated that IOP could be reduced by using GL. But even with the administration of GL, IOP was still higher than the controls. That result may be associated with these factors: 1) GL dosage; The usually used dosage for adults was 2 or 3 tablets per time and 3 times per day (150–225 mg GL per day). In this study, the dosage for rabbits is half tablet per time and 2 times per day (25mg GL per day). That dosage was calibrated on the comparison of body surface (rabbit versus human). But the rabbits could only receive two feedings per day according to their habits, not like human (three times per day). That difference may bring about insufficient GL concentration in ocular tissue. 2) TA dosage: The appropriate TA dosage for rabbit is 0.1ml/1.3 mg [28]. The high dosage we used here was just to make OH models. Even IOP in the GL-treated groups was lower than that in the TA group during the follow-ups; it was still higher than the controls. This result suggested that GL could not inhibit TA-induced OH completely because of the high TA dosage. 3) Species difference; Despite NZWR is a suitable model for 11β-HSD1 study, the species difference may affect the susceptibility to GL.

ERG represents the general function of the retina, especially the function of the photoreceptor and bipolar cells. Scotopic ERG mainly expresses the function of the rod cells, and photopic ERG expresses the cone cells. VEP response represents the general function of the optic nerve. In this study, the amplitudes were decreased and IT was prolonged in the TA group both in flash ERG and VEP during the follow-up, which indicated the retinal and optic nerve function was impaired. GL intervention had a protective effect resulted in better parameters in electrophysiology, which may also contribute to 11β-HSD1 inhibition.

The metabolism is necessary to maintain biologic activity of the cells (e.g., signaling and energy transferring between the cells). So the external influences (nutrition, drug, etc.) on cells can be better evaluated by analyzing their metabolites [23,25,26]. The pathogenesis of CIG/OH still remains unclear, but it is certainly in connection with glucocorticoid metabolism. So we used metabolomics here to study the glucocorticoid effects on ocular metabolism.

Compared to the controls, the significantly changed metabolites in the TA group at 1 day post-injection (represents the acute reaction phase after injection) are as follows: lipid (low density lipoprotein, very low density lipoprotein, choline, and glycerol), protein (isoleucine, leucine, valine, and alanine), sugar (lactate, acetate, and glucose), acute phase protein (N-acetylglycoproteins) and anti-oxidant (taurine). At 28 days post-injection (represents the chronic reaction phase after injection), the changed metabolites included lipid (glycerol), protein (alanine), sugar (lactate, acetate, and glucose), and anti-oxidant (taurine). Those data showed that TA could alter ocular metabolism in a time-dependant manner. Analyzed by PCA (Figure 2), the major biochemical difference between the TA group and the controls is that TA affects sugar metabolism, especially suppresses tricarboxylic acid cycle. That effect could lead to higher glucose level in aqueous humor, which could induce more mucopolysaccharide and fibronectin deposition in trabecular meshwork to reduce aqueous outflow [29-31]. This is the first time to announce that suppressed tricarboxylic acid cycle is the major biochemical reaction of CIG/OH.

Compared to the TA group, the changed metabolites in the TA+GL group and the GL+TA group post-injection are as follows: lipid (low density lipoprotein and very low density lipoprotein and choline), anti-oxidant (taurine) and sugar (lactate and glucose). The major biochemical change is that TA-induced effect (tricarboxylic acid cycle suppression) was partly inhibited by GL intervention. All those changed metabolites are closely related to pathogenesis of glaucoma: low density lipoprotein can induce mRNA overexpression of fibronectin, α-laminin and collagen type IV, all of which are participated in CIG pathogenesis [32]. Lactate is essential to ATP production and is important to excitatory neurons [33], the deposition of lactate represents that the neurons are damaged because of ischemia [34]. Choline is a key component of lecithin and sphingomyelin, and is useful in glaucoma treatment. Parisi et al. [35,36] treated glaucoma patients with citicoline and found that ERG and VEP was improved. That finding is also supported by other studies [37-40]. In this study, all those metabolites were significantly changed after administration of GL, which maybe is the metabolic mechanism of GL hypotensive effects.

11β-HSD1 is regarded as a potential target to regulate GC activity. In this experiment, GL, an inhibitor of 11β-HSD, was administrated in the TA-induced OH rabbits. Our data showed that GL could significantly decrease IOP in OH cases. The metabolomics study indicated that GL could affect TA-induced ocular metabolism. So according to our results, we hypothesized the following possible mechanism: TA could induce the changes of ocular metabolism, which may cause IOP elevation. GL, an inhibitor of 11β-HSD1, may block the excess production of cortisol by binding 11β-HSD1. Thus it could decrease IOP by compensating the changes of TA-induced ocular metabolism. Further studies in molecular biology are needed to prove that hypothesis.

11β-HSD2 was found in corneal endothelium or non-pigmented epithelium (NPE) of the ciliary body, while the mineralocorticoid receptor was present in the NPE cell line [18,41]. Those findings suggested that 11beta-HSD2 may play an important role in producing aqueous humor. Glycyron is a non-specific inhibitor of the 11β-HSDs, so the type 2 enzyme in the ocular tissue is also inhibited in this study. This isozyme protects the mineralocorticoid receptor from cortisol excess [42]. Switching off 11β-HSD2 could result in the changes in extracellular sodium, which may increase the IOP. The IOP changes after GL administration were depended on the overall effects of 11β-HSD1 versus 11β-HSD2: If 11β-HSD1 inhibition was stronger than 11β-HSD2 inhibition, the IOP would decrease, vice versa. Our results (IOP in the GL group was lower than the controls) suggested that 11β-HSD1 inhibition was stronger than 11β-HSD2 ones.

In a conclusion, the administration of GL could suppress OH induced by TA in rabbits (GL pre-treatment has better IOP control), and improve their electrophysiological parameters. Metabolomics is a useful tool in ophthalmology research. Our results indicate that TA-induce ocular metabolism changes could be compensated by GL.

We also acknowledge that there exists the potential for misleading conclusions, because the number of rabbits and the range of dosage are limited. And the molecule mechanisms still remain unclear. Further investigations are necessary to determine the efficacy of GL.
